# Narrative inquiry on early-career teachers’ stories of *Pagdadala* in caring for students in low-resource urban public schools

**DOI:** 10.1080/17482631.2021.1917881

**Published:** 2021-05-02

**Authors:** Ross Laurenne G. Fortunado, Nico A. Canoy

**Affiliations:** Department of Psychology , Ateneo De Manila University, Quezon City, Philippines

**Keywords:** *Pagdadala*, cost of caring, emotional labour, student wellbeing, teacher wellbeing

## Abstract

Purpose: In low-resource public schools, these costs may be amplified for early career teachers who help students bear increasingly complex burdens despite lack of resources and specialized support. However, there are limited studies on how care work and its costs are experienced by early-career Filipino public school teachers in low-resource contexts. Hence, the purpose of this study is to examine teachers’ stories of caring for burdened students using an integrative and critical narrative inquiry based on Clandinin’s narrative framework and Decenteceo’s cultural story-model of *Pagdadala*(i.e. burden-bearing). Methods: Field texts were collected through in-depth interviews with ten (10) female teacher advisers over two months. Participants came from eight (8) different public schools catering to students from low-resource communities in Marikina City, Navotas City and Quezon City. Results: Findings showed four narrative pathways ofpagdadalaof caring that teachers lived and told across the caring landscape: shared, overextended, asserted, and curtailed. These non-linear pathways reflect how teachers’ experience of care work is shaped by the overlapping sphere of influence of homes, schools and communities in student care. Conclusions: Complimenting literature on care work in education using Clandinin’s narrative inquiry framework that integrated Decenteceo’sPagdadalamodel, this study has offered a storied map of co-burden-bearing that was shaped by the social, spatial and temporal contexts in low-resource urban public schools. Theoretical and practical implications highlight the dynamics of bigat-gaanin care work and the potential advantage of leveraging on sharedpagdadalaand spaces of pagpapahingain supporting teacher wellbeing.

In low-resourced public schools, teacher wellbeing is beset by complex work demands, including the amplified responsibility of caring for students. The teaching profession has long been known for its high levels of occupational stress and burn-out (Acton & Glasgow, 2015). In low-resourced contexts, teacher stress is further linked with exposure to poor working conditions such as limited material and human resources, high student-teacher ratio, work task overload, behavioural difficulties of students, managing multiple roles, and low salaries (García-Carmona et al., 2019; Montgomery & Rupp, 2005). While distressed learners are entitled to receive specialized psychosocial support in schools, the lack of such resources in these contexts inevitably places the burden of caring on the teachers’ shoulders (Denise Valdez, 2018). Teachers have supported distressed students in a variety of ways, from providing more general academic, socio-emotional, financial and physical support, up to implementing more specialized school-wide psychosocial interventions (Franklin et al., 2017).

While the teachers’ caring role significantly benefit learners’ holistic development, it can also entail psychological costs like experiences of emotional heaviness or *bigat* in *pagdadala* for teachers (Alisic et al., 2012; Decenteceo, 1997; Koenig et al., 2018). The Filipino concept of *pagdadala*, based on the cultural story-model of *Pagdadala*, uses the locally-embedded metaphor of bearing the burdens of everyday responsibilities and cope with life challenges (Decenteceo, 1999). In teaching, we posit that “co-bearing” the burdens of students from low-resource contexts, especially those in distress, can also intensify psychological costs for educators such as feelings of stress, role confusion, compassion fatigue and burn-out (Abraham-cook, 2012; Figley, 1995). However, despite what is known about the importance and potential risks of teachers’ caring for burdened students, there are few empirical studies that illuminate how this experience unfolds for early-career teachers in low-resourced contexts (Koenig et al., 2018).

As such, this study appropriated Clandinin et al. (2016) Narrative Inquiry framework to explore early-career teachers’ lived and told narratives of *pagdadala* of caring in low-resourced public schools. This framework assumes that “humans, individually and socially, lead storied lives, and subsequently shape their daily lives by stories of who they and others are as they interpret their past in terms of these stories” (Clandinin, 2006 ; p. 375). This paper aims to contribute to literature on care work with burdened students by offering an emic narrative framing that considers not just individual storied experience of teachers, but also how their stories are shaped by the socio-cultural and spatial contexts where they occur in time. It also aims to contribute more empirical grounding in how Decenteceo’s cultural story-model of *Pagdadala* can be used as a theoretical scaffold to understand culture specific burden-bearing experiences of early-career teachers and inform the design of relevant psychosocial support interventions and care-based policies in low-resourced contexts.

## Literature review

### Teacher care and students’ academic success and wellbeing

Teachers support students in varied ways that positively promote their academic success, moral development and socio-emotional development. This may include personal care, or attending to students’ socio-emotional problems to promote their identity and dignity as a person, and academic care, or pedagogical activities that bring about learning in the classroom (Noddings, 1992; Wentzel, 1997). Teachers’ emotional support has been linked with students’ increased engagement in school, emotion regulation skills, and better coping skills (Jennings & Greenberg, 2009; Wentzel, 1998). Their social support is linked with positive academic emotions, sense of connectedness and modelling of pro-social behaviour (Jennings & Greenberg, 2009; Lei et al., 2018). Teacher-facilitated psychosocial interventions endorsed by trauma-informed educational approaches is found helpful in buffering trauma-effects in students (Franklin et al., 2017). In low-resource contexts, teachers may emphasize discipline, material assistance and physical support as caring versus more emotion-centred care endorsed by western policies (Coultas et al., 2016).

While the teacher’s caring functions are beneficial for students, especially those in distress, they come with psychological costs for the educator. Studies suggest that caring for struggling students affect teachers’ construction of identities and meaning of their work. Teachers who successfully address students’ needs found care work as personally and professionally meaningful (Brunzell et al., 2018) and have increased sense of compassion satisfaction, professional competence and commitment (Abraham-cook, 2012; Hill, 2011). However, teachers’ lack of specialized psychosocial care knowledge may lead to role confusion, blurring of professional boundaries, and foster a sense of incompetence (Alisic, 2012). Failing to address students’ needs also led to lowered sense of professional competence and is taken hardest by teachers who highly valued caring roles (Hill, 2011).

Western studies highlight a range of cognitive and emotional distresses teachers experience when helping distressed students. To effectively care for students, teachers may engage in emotional labour and experience dissonance over needing to express emotions that is starkly different from how they genuinely feel (Wang et al., 2019). This includes suppressing their own distresses and projecting positive feelings to be able to provide emotional support to students. Compassion fatigue literature posit that teachers may vicariously experience the trauma symptoms of pupils like having intrusive thoughts regarding their students’ distress, feelings of helplessness and negative shifts in viewing the world (Figley, 1995; Koenig et al., 2018). Burn-out literature have described teachers to experience emotional exhaustion, depersonalization, and lowered sense of accomplishment with prolonged exposure to difficult circumstances (Abraham-cook, 2012). These negative effects of exposure to students’ sufferings appear to be influenced by the perceived intensity of students’ distress, teachers’ own experience of trauma, the length of teacher exposure and the lack of supportive systems for the teacher (Gu & Day, 2013; Ziaian-Ghafari & Berg, 2019).

### Caring for burdened students as co-burden-bearing

Experiences of cognitive and emotional distress in caring for others can be related to the local concept of *bigat* in the *Pagdadala* counselling model of Decenteceo (1996, 1999). This cultural counselling model views an individual’s experience of carrying out deeply valued responsibilities and bearing life’s struggles via the story-metaphor of burden-bearing or *pagdadala*. It has the following elements: the *taga-dala* (or burden-bearer who go through the journey of carrying responsibilities), the *dinadala* (or burden, task, responsibility or relationship that is carried by the burden-bearer out of a sense of duty), the *pinagdadaanan* (or path the burden-bearer goes through in carrying the burden), and the *patutunguhan* (or destination or conditions where the burden-bearing is directed). In this study, *bigat* in *pagdadala* pertains to the sense of heaviness and difficulties that one experiences in bearing one’s responsibilities.

Teachers as *taga-dala* can be seen as both burden-bearer and co-burden-bearers. Co-burden-bearers, according to Decenteceo, are helpers who are themselves *taga-dala* with their own burdens, but also bear the additional responsibility of helping other *taga-dala* carry their own burdens. The teacher co-burden-bearer’s *dinadala* is their responsibility of caring for their student burden-bearers. Their *pinagdadanan* consists of events and paths that they go through to achieve their aspired *patutunguhan* or aspired end-state for their students. The educator’s experience of *bigat* in *pagdadala* may involve the affective, cognitive and behavioural dimensions they relate with caring for students. Aside from *bigat, Pagdadala* also considers having *gaan* or sense of lightness and ease. Decenteceo recommends that taking *pagpapahinga* or taking a breath out to ease the process of burden-bearing.

### Need to care for teacher-carers

Despite what is known about the benefits and risks of teacher’s care work, there are few empirical studies that illuminate how this experience unfolds for early-career public school teachers in low-resource contexts. While studies on promoting struggling students’ wellbeing via teacher care abound, promoting teacher wellbeing as they engage in care work has only begun to receive attention (Wessels & Wood, 2019). Majority of literature on teacher care work with distressed students also hail from western contexts where teachers have greater access to specialized psychosocial support manpower and school-wide programmes. In this study, we focused on how Filipino urban public school teachers contend with the increasingly complex psychosocial concerns of students in low-resourced school contexts.

In the Philippines, for instance, while the implementing rules and regulations (IRR) of the Philippine Mental Health Act (Official Gazette of the Republic of the Philippines, 2019) called for an increase in quality and access of specialized psychosocial support in schools, there remains a pervasive lack of the material and human resources and enabling support systems that can aid ease of implementation (Denise Valdez, 2018). Nationwide, there are only over 3000 licenced guidance counsellors vis-à-vis the 22-million public school learners, leading to the creation of “guidance teacher” roles^1^ filled-in by educators (Denise Valdez, 2018; Department of Education, 2015). In this specific context, public school students may experience added distress over poverty-related challenges as well as higher vulnerability to community violence, calamities, health concerns, and rising cases of youth mental illness and suicide cases (Geronimo, 2013; Tomacruz, 2019).

The challenge in caring may be exacerbated by the sheer number of students that teachers handle in over-crowded classrooms or multiple class shifts, alongside the high volume of administrative paperwork and multiple other support roles (Malipot-Hernando, 2018). Urban public school teachers also report being stressed by student’s misbehaviours vis-à-vis a perceived lack of available sanctions for misbehaviour because of child protection policies (Pagayanan, 2016). All of these maybe especially heavy for early-career teachers who are still learning the ropes of their roles, and tend to leave teaching within the first five years of teaching due stress (Schaefer & Clandinin, 2018).

Given these challenges, there is a need to explore how early-career public school teachers experience and can be supported in their care-related work. While *Pagdadala* may be a culturally-relevant model to understand care work and design relevant interventions to mitigate its costs, there are limited studies on its application to the education context. Current *Pagdadala* literature are chiefly based from Decenteceo’s own reflections of his clinical practice with advocacy workers (Decenteceo, 1997, 1999), or more recent application in studies of children’s experiences in conflict areas (Noguera, 2013) or psychological first aid with disaster survivors (Landoy et al., 2015). Mapping early-career public school educators’ stories of *pagdadala* can inform the design of culture-specific psychosocial interventions for teachers who support struggling students.

### *A narrative inquiry into teachers’ experiences of* pagdadala *in caring*

In this paper, we specifically used Clandinin’s Narrative Inquiry (Clandinin, 2006) to map out teachers’ experiences of *pagdadala* in caring. Since *pagdadala* was derived from a story-metaphor model, mapping this experience using a narrative framework was deemed appropriate. Clandinin’s narrative inquiry views “stories as ways by which individuals enter and make sense of the world” around them, as storied experiences unfold in the three-dimensional commonplaces of sociality, temporality and physical space (Clandinin et al., 2016). Sociality pertains to personal conditions of the narrator (feelings, hopes, desires, aesthetic reactions, and moral dispositions of both participant and inquirer) and their social conditions (environment, surrounding factors and forces, and people that form a person’s context). Temporality refers to the narrator’s sense of continuity, as experiences continuously unfold across past, present and future. Lastly, spatiality pertains to the physical spaces and situations where events unfold.

Clandinin’s Narrative Inquiry framework considers how storied experiences are shaped by the context of the individual, and the social and cultural space where their experience unfolds. Furthermore, this framework also anchors on a relational approach to research, encouraging the co-creation of meanings between the researcher and the participant (Clandinin et al., 2016). Through Clandinin’s narrative inquiry framework, we aimed to answer the following main research question: “What narratives of *pagdadala* in caring for students do early-career teachers in low-resource public school live and tell?” In particular, we ask:

1. How do teacher-carers map their identities as the *taga-dala*?
  - How do they define their roles as they provide care for students in need?
  - What meaning and purpose *(patutunguhan)* do they ascribe to their caring function?
2. What characterizes the teachers’ stories of *pagdadala* in caring?
  - How do they engage in the *pagdadala* of their caring responsibilities?
  - What thoughts, feelings and actions do they associate with their experience of *bigat* and *gaan* in their *pagdadala* over time?
3. In which spaces and situations do teachers engage in *pagdadala* and *pagpapahinga*?

## Methodology

### Participants

Field texts were collected through in-depth interviews with ten (10) female teacher-advisers over two months. Participants came from eight (8) different public schools catering to students from low-resource communities in Marikina City, Navotas City and Quezon City. Targeted schools catered to two class shifts per day to accommodate the high volume of students, had no dedicated licenced guidance counsellors, and were located in the top 8 Metro Manila cities with the highest numbers of depressed households (United Nations—Habitat, 2003). Participants were recruited using a combination of purposive and snowball sampling through online recruitment advertisements and referral requests from our network of public school teachers in targeted areas. The predominance of female participants reflects the trend in the Philippine teaching workforce with over 85% female (Olores-Regalado, 2017). A male teacher was initially recruited for the study but was eventually excluded due to wellbeing challenges. Potential participants were invited separately and sent soft copies of the Informed Consent Form with details on the overview of the study, the wellbeing screening tool, participant qualification requirements, and Php500.00 gift certificate participation incentive.

#### Wellbeing screening

To minimize the wellbeing risk posed by sharing potentially distressful experiences, only participants with positive levels of wellbeing were included in the study. To determine their current level of wellbeing, potential participants were requested to take the clinical stress tests Depression, Anxiety and Stress Scale or DASS-21 (Lovibond & Lovibond, 1995) and the WHO-5 Wellbeing Scale (Topp et al., 2015). The author trained in Counselling Psychology individually administered the tests in venues that participants chose as most convenient for them (e.g., quiet spaces in their homes, schools or establishments near the school). Out of thirteen initial potential participants, one was excluded from the study due to very low wellbeing levels and was provided assistance for a free initial mental health consultation with a psychosocial support centre (fee is shouldered by the author). Two others were excluded as their tenure went above five years.

The ten teacher-participants had an average age of 26, an average of 3.5 years of public school teaching experience, and at least one month of private school teaching experience (see Table I). All were licenced educators with no psychology-related degree or licensures. All held advisory roles for at least a year and had job descriptions which integrated psycho-social care functions for students (e.g., guidance and counsel provision, being second parent, holding advisory classes, and coordination of student concerns). Seven teachers spend their whole day with their Kinder, SPED and lower grade self-contained^2^ classes, while the rest spend Advisory period and taught at least one other subject for their upper grade level students. Beyond their advisory functions and teaching load, all teachers also had other extra-curricular responsibilities to attend to after or between class hours. Only three of the teachers originally reside in the communities where their schools are, while the rest are from other Metro Manila cities or provinces beyond the region.

**Table I.** Demographic information

Participanta	Age	Position and Roles	Grade Level Handled	Years in Public School as of February 2020	City of School
Queena	22	Adviser (self-contained), GSP, Reading Remedial	Grade 3, Grade 5	1 year and 9 months	Marikina City
Daisy	23	Adviser (self-contained), Employment Coordinator	SPED	1 year and 10 months	Marikina City
Elaine	23	Adviser (self-contained), MTB-MLE, Reading Remedial	Grade 1	1 year and 6 months	Marikina City
Me-ann	25	Adviser, Reading Remedial, ICT Coordinator	Grade 5	2 years	Navotas City
Lucy	26	Adviser (self-contained), vocational/perceptual skills	SPED L-3	4 years	Quezon City
Anna	26	Adviser, Science Teacher, Clinic Teacher	Grade 5	4 years and 6 months	Marikina City
Jenny	27	Adviser, Reading Remedial, Club Adviser	Grade 6	4 years	Navotas City
Vanessa	27	Adviser (self-contained), Reading remedial	Grade 1	5 years	Marikina City
Bhe	30	Adviser (2 classes, self-contained), Reading Remedial	Kindergarten	4 years	Navotas City
Shay	33	Adviser (self-contained), ESP Coordinator, Researcher	Grade 1	4 years and 11 months	Navotas City

a Participant names are altered to ensure confidentiality

### Data collection procedure

Ethics clearance was approved by the [redacted for peer review] before implementing the protocol. Participants were interviewed for 1 to 3 hours in venues most convenient for them, such as their homes, inside their classrooms, or in establishments near their school. The key narrative question in the interview involved asking teachers to share their most salient story of caring for students: *“Without mentioning students’ names, can you share a story about your most salient experience here in school where you had a student who was going through a challenge or had heavy burdens to bear and you were the one who supported or cared for them most? You can start with how it began, what you did when you found out, and how it concluded.”*

Probing questions were asked to contextualize participants’ narratives and clarify details on how participants identified themselves as carers, how they experienced *bigat* or *gaan*, and spaces where they engaged in *pahinga* or rest. With participants’ consent, all interviews were audio-recorded and pertinent context observations were noted on interview schedule sheets. Recorded interviews were transcribed, re-checked for completeness, and added with notes on participant’s feelings or gestures, when necessary.

Since Clandinin’s narrative inquiry placed emphasis on the quality of relationship between the researcher and the participant, space was made for more informal conversations and interactions before or after the interview proper (e.g., eating at the canteen where Teacher Daisy’s SPED students served as staff for their livelihood skills, chatting about non-interview related topics, etc.). After the interview proper, participants were asked how they felt about sharing their stories to ensure that the story-sharing did not bring unnecessary distress. Refreshments and incentives were provided for all except for three teachers who explicitly refused material incentives. Before or after each interview, we recorded our thoughts, feelings and observations about the research puzzle or the interviews on a field notebook or electronic voice notes.

### Data analysis

Data analytic process followed an integration of insights derived from Clandinin and Conelly’s narrative inquiry and Decenteo’s *Pagdadala* model. First step, Clandinin and Connelly suggests three analytical tools for narrative inquiry: *broadening* (i.e., looking for the broader context of the story), *burrowing* (i.e., paying attention to the participants’ feelings, understandings or dilemmas or certain events’ impacts on the participants or surroundings), and *storying and restorying* (i.e., finding ways to story and re-story data so that the significance of the lived experience of the participant comes to the fore) (Kim, 2016). Second step, these guidelines were followed in re-storying participants’ field texts into individual narrative accounts whose format highlighted key elements of Decenteceo’s *Pagdadala* model. Third step, we aimed to achieve conceptual clarity and thematic coherence by composing an overarching storyline linking different elements of Decenteo’s model. The storyline is as follows: “As a teacher-carer I am … (integration of participants’ responses pertaining to their sense of identity as a caring teacher or the co-burden bearer/*taga-dala*), bearing the burden with … (challenges and context of student cared for or the *dinadala*), whom I cared for by … (processes engaged to carry out their caring responsibility or *pagdadala*), through … (the day-to-day events they went through in their narratives or their *pinagdaanan*), so that … (their perceived end-goal/purpose for their caring actions or perceived *patutunguhan*). Throughout this story, I personally experienced *bigat* as … (evaluation of how heavy/light their experience of *pagdadala* was or their experience of *bigat/gaan)*”.

Fourth step, themes for the final research text were drawn by thematically analysing each narrative account to see wider patterns or paths in how participants identified themselves as *taga-dala* and related with other characters in their caring stories (sociality), how teachers cared for their students and experienced the *bigat* or *gaan* of caring across time (temporality and sense of continuity), and where the teachers’ experiences of *pagdadala* took place (spatial). In this interpretative step, we recognize that not all participants fell uniquely into a single path (i.e., one participants can fall under multiple paths) or that my labels don’t quite fit the participants’ experiences. Hence, we engaged further validation in the fifth step.

Fifth step, research validity of the study was strengthened by including Clandinin’s touchstone of the co-creation of the interim texts with the participants and having a relational response community (Clandinin et al., 2016). A *relational response community*, composed of individuals other than the first author that have some degree of stake to the research puzzle and are willing to read, respond and co-compose field texts, interim texts, and final research texts (Clandinin et al., 2016), was recruited. Members of the relational response community included three former public school teachers who taught in targeted communities, a current public school teacher advocating for teachers’ mental health, a teacher trainer, and the second author. To maintain confidentiality, members of the relational response community were given access to data that bore no identifying information about participants.

In terms of reflexivity, the co-construction of the *pagdadala* narratives of participants was significantly shaped by the first author’s previous experience as teacher and guidance officer in a low-resource rural school, wellbeing coach and trainer of public school teachers, and academic background in counselling psychology. In order to maintain critical distance, iterative feedback and consultations were conducted with the second author who is a university-based teacher and social psychologist. Throughout the analytic process, both authors draw from their own experiences of co-burden bearers, albeit in a different context, to simultaneously empathize and question the narratives co-constructed with the participants.

Since the timeline of validation for this study occurred during Metro Manila’s first month of the Enhanced Community Quarantine (ECQ) against COVID-19 (Al Jazeera, March 2020), the validation step on co-creating interim and research texts with participants was modified. Interim narrative accounts and initial results of the study were electronically sent to the participants for transparency and for their validation and feedback. It was reiterated, however, that they may respond only if they can, as they may be pre-occupied with the current crisis. Participants were also electronically messaged to check-in on how they were doing during ECQ. Eight out of ten participants were able to provide validation and feedback to further refine the results. In the final write-up, Filipino words or phrases derived from Decenteceo’s original model was retained to maintain the cultural integrity of concepts.

## Results

Decenteceo’s *Pagdadala* story metaphor of a co-burden-bearer journeying along the *pinagdadaanan* to carry the *dinadala* towards a *patutunguhan* is borrowed in framing the results. Re-storying the teachers’ narratives using these elements yielded four different narrative pathways or *pinagdadaanan* in the *pagdadala* of one’s caring responsibility: shared *pagdadala*, overextended *pagdadala*, asserted *pagdadala* and curtailed *pagdadala*. Embedded in dynamic spaces of schools, homes and communities across time, each pathway featured the salient ways by which teachers as *taga-dala* lived out their roles, *patutunguhan* and ways of caring for burdened students. The pathways highlight how the teachers’ roles, *patutunguhan* and ways of caring were shaped in varying degrees not just by teachers’ interaction with the students per se, but also with interactions with the students’ other carers (e.g., guardians, parents, co-teachers) and caring systems across spaces of care. The pathways also featured how teachers experienced *bigat* and *gaan*, along with how and where they are able to engage in *pagpapahinga*, in the course of their *pagdadala* of care. Identifiers used for teachers and students in the succeeding narratives are pseudonyms.

### *Caring along connected paths: shared* pagdadala

Stories of shared *pagdadala* featured teachers who bore the responsibility of caring for students primarily by establishing meaningful connections with them and even their guardians. Teachers identified as students’ second parent and the guardian’s partner in caring, hence becoming a co-burden-bearer for both. The *patutunguhan* in shared *pagdadala* highlighted the attempts of the teacher to establish a shared inner connection with their students, as well as closely partnering with guardians across affiliative spaces of care. Since the teacher and guardian share the same path and *patutunguhan* in caring for the student, the burden of responsibility is divided between more co-burden-bearers and the sense of *bigat* was experienced as *magaan* through time.

#### “Lola, let’s educate lucky together*”*

Shared *pagdadala* is illustrated in Teacher Daisy’s story of caring for Lucky, her fifteen-year-old SPED student who had cerebral palsy and wanted to be a waiter for an on-the-job training (OJT) stint. As a SPED Advocate, Daisy endeavoured to identify her SPED students’ potentials and help them fulfill it. In their months together, however, Daisy found it challenging to deal with Lucky’s disruptive behaviour and frequent absences. By visiting Lucky’s house to interview and involve his guardians in designing his education plan, Daisy discovered that Lucky’s misbehaviours stemmed from the pain of his mother’s abandonment. She also discovered that it was the grandmother who discouraged Lucky from going to his OJT opportunity for fear of people mocking his disability.

Attempting to be a second mother to Lucky, Daisy listened to Lucky’s stories in private moments in her classroom. She afterwards shared updates with his grandmother to help her deal with Lucky’s issues at home. Daisy reassured her, “*Lola*, don’t be embarrassed. Let’s educate Lucky together.” Daisy also assigned Lucky as cashier at their OJT site because of his average intelligence and publicly praised him in social media for his work. Appreciative of this, Lucky’s guardians gradually became more optimistic about his work prospects and encouraged him to attend school more. Reflecting on her collaboration with guardians, Daisy shared that it shaped her identity as a SPED advocate and deeply connected her with their families:

“At first, it’s like, my focus is (with) PWD. But now, because of the parent’s stories, it’s like I also wanted to empower them. Hearing their life stories (…), it’s like becoming a family. I love my students, but I also have to love—especially I have to love the parents because they will support my students when I am gone.”

#### Affiliative spaces of care

Similar to Teacher Daisy’s story, other shared *pagdadala* narratives were characterized by enabling parent-student-teacher collaboration occurring across affiliative spaces of classrooms and homes. As second parent inside their classrooms, teachers utilized their *pakikiramdam* (shared-inner-perception) to connect meaningfully with their students, assess signs of distress, discern sources of misbehaviour, and discipline them. Teachers often described pivotal moments of connection which marked transitions from difficult to easier days in managing especially disruptive students. One-on-one check-ins were conducted in private at teacher’s table or empty classrooms where teachers attended and responded to students’ needs—be it helping “process” students’ difficult experiences, explain a disciplinary action given, offer advice, or agree about action steps.

Teachers tended to discover the student’s *dinadala* as rooted in their home spaces where guardians also lived their own caring narratives for their children. Hence, beyond school, teachers became the parents’ partner in addressing student concerns at home. Guardians and teachers discussed concerns, problem-solved and agreed on next steps together. They exchanged stories in affiliative spaces of care such as parent-teacher meetings in school, home visitations, or in parent-teacher chat groups and text messages. In some instances, like Teacher Queena’s sharing below, parents even became co-burden-bearer of teachers in their school work, lending to parents’ deeper understanding of their children’s lives in school:

“I said [to the parents], can you come to the school and look over [the students] because I have to do something? So the parents came to look over the students, and they saw how rowdy they were. [They realized] ‘Ah, so it’s true that there are students like these.”

#### Bigat *and* gaan *of connectedness*

The heavy responsibility of caring in shared *pagdadala* was eventually felt as more *magaan* over time because of the rewards of connectedness and collaboration. Valuing caring as an essential part of teaching, *bigat* in shared *pagdadala* centred more on the internal pressure teachers put upon themselves to carry their duty well, as they also juggled the burdens of their personal lives and administrative responsibilities, as Teacher Me-Ann expounded below.

“When you enter the classroom, you handle so many lives. (…) If you’re the kind of teacher who has real concern for students, you really carry it with you. So, aside from your personal problems, aside from your home problems. You really carry within you the problems inside the classroom. On top of added work from the principal.”

Sometimes, *bigat* was also felt when teachers inadvertently become confidants to parents’ non-student related troubles, such as parent-to-parent conflicts, domestic concerns, etc.

Meanwhile, *gaan* included the sense of ease enabled by a divided and delegated care-related workload between guardians and teachers. Aligning goals and ways of caring helped reinforce disciplinary approaches across school and home. Teachers worried less about distressed students, reassured that parents will take care of them at home. *Gaan* also related with the sense of fulfilment when shared *pagdadala* led to resolve students’ difficulties, as well as the inherent joy of positively connecting with students and their families. Teachers Queena and Me-Ann elaborated on this sense of *gaan* related to success and positive connection:

“The children themselves make things lighter. Aren’t they my source of joy?” Teacher Me-Ann

“It’s easier because both of us now provide discipline. (…) That’s where I got the type of parent support where, the type where children do their assignment [at home]. And I would see in class that the children help each other. And the beauty of that is (…) my parents have gotten to know each other too.” Teacher Queena

Aside from finding joy in students in the classroom, spaces of *pagpapahinga* included places where teachers can detach from school, engage in self-care and celebrate their hard work:

“Sometimes, when you are too focused on your students, you forget yourself. (…) You also need to treat yourself, really. (…) I’ll go to the mall, eat, go into the salon. That’s what I do to self-treat myself. ‘Oh, because you did well this week, you were productive,’ I tell myself.” Teacher Me-Ann

### *Caring along neglected paths: overextended* pagdadala

Overextended *pagdadala* narratives featured teachers who bore their responsibility of caring for students by filling-in the material and socio-emotional needs that guardians or the school inadequately provided. Since the care provided by guardians and school support systems are perceived as deficient or absent, the teachers identified with supplementary roles such as pseudo-parent, friend, clinic teacher and others. To reach the *patutunguhan* of supplementing what is lacking and sustain students in school, students were supported in over-extended spaces of care. Experience of *bigat* in this narrative included feelings of distress over the student’s disadvantaged situation and the strains of providing supplementary help. *Gaan* was related with a teacher’s sense of fulfilment in seeing positive impact of one’s support, managing workload better and receiving support for themselves as caregivers.

#### “I’ll provide your allowance”

Overextended *pagdadala* is illustrated in the story of Teacher Bhe with her former first grader Ben-ben, a diligent student whose parents could barely provide for his schooling. Because she grew up with adoptive parents and knew what it was like to be deprived of care, Bhe endeavoured to give to students “the care you also want to receive”. Initially, she was annoyed at how Ben-ben would sometimes come late and wet for school. But Ben-ben explained later, “Cher, I swim because I don’t have money [to pay for the boat].”

Bhe was so shocked about Ben-ben’s situation that she promised to provide for his daily allowance, “I’ll be the one to provide your allowance, as long as you come to school.” She also sometimes gave money to Ben-ben’s parents. Ben-ben was eventually able to move up to higher grades and was already in the sixth grade at the time of the interview. Teacher Bhe reflected on Ben-ben’s story as one that always inspired her to become a better teacher:

“I cry when I remember this. I told myself, I want to be a more capable teacher. Someone who can give whatever I can still give—so they won’t feel inadequate. I have one student who now goes to school every day. He said, ‘Teacher, whatever happens, I’ll go to school.’ My other student said, ‘Teacher, because I learn from you.”

#### Overextended spaces of care

Like Teacher Bhe, teachers with stories of overextended *pagdadala* go beyond what is required and make up for the deficiency of other co-burden bearers or caring systems in overextended spaces of care. Inside the classroom, teachers listened to their students’ stories of neglect, abuse, hunger, or poverty and often came to understand that unless these material and socio-emotional needs are met, learning would not occur in school. Often, their attempts to share *pagdadala* did not work because guardians were either unresponsive, incapable or simply absent in their children’s school affairs. Hence, beyond just an academic space, teachers converted their classrooms into spaces where students found respite from home stressors or material provisions such as food, money or school supplies coming from teacher’s own pockets. For students with perceived unmet socio-emotional needs, teachers took on roles of being a pseudo-parent, sister or friend, or found creative ways to integrate socio-emotional support in their curriculum like having life-story sharing time. This was what Teacher Lucy did for her first and most difficult Grade 3 class upon discovering their impoverished situations:

“It’s hard to handle when you get angry as a teacher, so I became a friend. (…) They became ok. That’s when I understood that – some of my students still need to work, like after school they go to the streets to beg. (…) Maybe here in school, they are kids. Like they express their youthful mischief because they are so stressed at home. Their parents make them work.”

When it is the school that cannot provide ample manpower to deliver caring-related services, clinics and guidance offices become the overextended spaces where teachers became clinic teachers and guidance teachers on top of their academic teaching load.

#### Bigat *and* gaan *of overextending*

The experience of *bigat* in narratives of overextended *pagdadala*, similar to other stories of co-burden-bearing, highlighted shock and great pity of teachers over the students’ disadvantageous situations. But this *bigat* also tended to be tinged with disappointment and frustration over guardian’s lack of support and how one’s care in school did not transcend to the students’ home life:

“That’s what makes it – makes it heavy for me. How can I help if this parent won’t help. What other help can you give when it’s like that – I’m consistent at school, how I wish it’s also like that in [their] homes.” Teacher Lucy

And since teachers generously give of their material, physical and socio-emotional resources for students, *bigat* was also associated with the strains of heavy workload or multiple and sometimes competing roles. Such is the case for Teacher Anna below who was often torn between being subject teacher and clinic teacher when medical emergencies arise:

“Sometimes I worry that something might happen [in the class I leave] when I go down [to the clinic]. Sometimes, I just say that I want to cut my body in half – so someone can overlook the class, then someone can go here [clinic]. But I can’t do anything because those are the roles I’m given, I’ll accept.”

Anna’s worry is exacerbated by the fact that teachers are held legally responsible for students under their watch. Accidents in an unattended classroom could lead to a legal case of negligence or even loss of professional licence. Overextension also sometimes led to depletion of teachers’ material or emotional resources, especially when their efforts were underappreciated:

“Honestly, I really cried in front of them. Because I was spent. I gave everything, everything they wanted. Of course as a new teacher you’re so idealistic and you provide – materials, staying up at night – I came to the point that I would stay up at night for your exams, quizzes, and then they didn’t take it seriously.” Teacher Lucy

On the other hand, *gaan* in overextended *pagdadala* is experienced by teachers when their generosity eventually contributed to positive outcomes for students, such as increased class attendance, finishing the academic year and moving up to higher grades. As with Teacher Bhe’s caring for Ben-ben, a successfully carried out overextended *pagdadala* often led to feelings of fulfilment in significantly contributing to the students’ success in school and life. The missing support from guardians or the school may be augmented by social support from co-teachers, supervisors or family members. Engaging in spaces of *pagpapahinga* where they exchange student stories and learn of best practices during informal time with co-teachers in corridors, canteens, and clinics were found helpful, as Teacher Anna shared:

“Since I’m in the clinic, they [co-teachers] come here, so we talk. (…) Usually we talk about our problems with student this or that. ‘Gosh, what do I do?’ Stuff like that. I overhear them. So if I know something, I share it. But most of the time I’m the one learning from them.”

Learning effective management of one’s inner resources such as emotion, physical energy and time was also cited as greatly helpful. Since teaching could be a physically and emotionally taxing work, being able to find spaces of *pagpapahinga* at school where one can rest awhile without interruption or resting at home without take-home work were found helpful in recharging used-up inner resources. Bhe shared how she would find ways to rest and re-charge in between her two kinder shifts:

“Sometimes I sleep in the next classroom because my co-teacher has a house [instructional material] there that’s vacant. Sometimes it’s like I need to rest, need to rest my brain.”

### *Caring along clashing paths: asserted* pagdadala

Teachers with narratives of asserted *pagdadala* are those who bore their responsibility of caring for students by taking measures that clash with the caring ways of other co-burden-bearers (e.g., guardian, co-teachers, and supervisors) or school mandated ways of caring for students. Teachers identified themselves as *naninindigan* or carers who stood their ground in clashing spaces of care despite threats of conflict or professional sanctions. The *patutunguhan* of their caring actions included putting students in the rightful path and model what is fair and just. The experience of *bigat* in asserted *pagdadala* highlighted teacher’s feelings of threatened personal and professional dignity, sense of indignity, and strained connections with co-burden bearers. Experiences of *gaan* were related to receiving validation for their asserted ways of caring, as well as maintaining hope for future change in systems.

#### “I’ll fight for this”

Asserted *pagdadala* is illustrated in Teacher Shay’s story of caring for her Grade 1 class and Ruel, a student who barely went to school and had failing marks for class. Teacher Shay believed that caring entailed putting students in the right path and enabling them to handle themselves independently in the future. Shay and Ruel’s mother initially agreed that repeating Grade 1 would be best for Ruel, given his low competency and lack of readiness for Grade 2. The parent, however, heard Shay’s co-teacher say that “the school doesn’t really flunk students” and complained at the principal’s office about Ruel’s promotion. Shay stood her ground even while the parent threatened to complain her at the Division Office, “Are you sure about what you’re telling me? I find that unfair for my students. They put effort—and here you have someone who just complained and they get to pass. I’ll fight for this.”

The principal called for an investigation of Ruel’s records and called up the other co-teachers whom the parent spoke with. It was an incident the whole school got wind of. The principal initially sided with Shay on the matter. However, after discussing the case with the Division Office, eventually decided to put the student through summer modular classes and still get promoted to Grade 2. A year after the incident, Teacher Shay narrated how “destructed” she felt after that time, and how it greatly affected her sense of dignity as a teacher:

“As a teacher, your credibility, your dignity, it’s diminished. Other teachers also lose the motivation to teach because no matter what I do, [students] still pass. Even if I don’t take work seriously, I’ll still get paid.”

#### Clashing spaces of care

Similar to Shay’s story, narratives of asserted *pagdadala* occurred in clashing spaces of care where different co-burden bearers asserted their differing ways of *pagdadala*, or where the teachers’ *pagdadala* was going against prescribed ways of caring by institutional policies or practices. In these spaces, teachers who identified as *naninindigan* asserted to live out their aspired narrative of caring amidst other conflicting narratives, with varying degrees of success. The classroom became a clashing space of care when teachers clashed with behaviourally disruptive students who challenged the teachers’ caring ways. Also when the teachers’ ways of caring or disciplinary styles clashed with parents who complain about the treatment of their children, or with school-mandated policies and practices. Teacher Shay below shared about Kiyong, her student who threatened to kill her with a pencil during an outburst, and whom she managed in ways that went against the Child Protection Policy (CPP):

“ … It got really physical and the threat was worded as ‘I’ll really kill you!’ (…) He went berserk. So I went berserk too. That got him to stop. Sometimes it’s like you need to do it, even though it’s not right or you’re not really like that. But in that situation, it’s needed. You’ll risk your license so that – so that the child will be set right.”

Other teachers shared how they found certain provisions of the CPP as limited in helping them deal with the toughest and most behaviourally disruptive students, especially during episodes where other students and teachers were in immediate danger. Most of these students, they observed, also tended to be the ones with highly distressful home contexts.

Meeting rooms and administrative offices became clashing spaces of care when conflicts occurred between the teacher’s aspired *pagdadala* versus the prescribed and lived *pagdadala* of co-teachers, supervisors and other figures of authority, who enforced compliance with caring-related policies or informal practices. Teachers asserted or defended themselves in places of administrative authority such as the Principal’s Office, the *Barangay* Hall or in the Division Office where official complaints or escalated disagreements about student practices are dealt with. In a meeting with co-teachers who were insisting on revising the grades of the students, Teacher Elaine shared how she volunteered for the grade coordinator’s role as a way to stand her ground against her co-teachers’ practices:

“I told them: ‘Ma’am, whatever you passed to me, that’s what I’ll report. I won’t defend you. I’ll only defend what I consolidated.’ That’s when I heard things like, ‘Oh, you’ll give us away?’ Not that I’m giving you away, but that’s what you passed.”

#### Bigat *and* gaan *of taking a stand*

The teachers’ experience of *bigat* in asserted *pagdadala* related to consequences of standing by their aspired caring narratives. This included a sense of indignity over situations where teachers tried to live out caring practices they believed to be right in the backdrop of practices and prescriptions they perceived as wrong or unhelpful. Teacher Jenny below echoed narratives of her co-teachers regarding the “practice of mass promotion” and assigning blame for students’ underperformance to “teacher factor”:

“That’s all they think about, that if a kid drops out, it’s the teacher’s fault. It’s degrading. (…) My co-teacher who handles Section 7 went to her student’s house to tell him to go back to school. The kid retorted, ‘Even if I don’t go back, I’ll still graduate.” Teacher Jenny

Asserted *pagdadala* narratives whose resolutions were not amicable to parties involved led to strained social relations afterwards. In situations that risked physical harm, antagonism, administrative sanction, or job loss, *bigat* was related with threatened feelings of physical safety, psychological safety and sense of dignity. Teacher Elaine elaborated on lessened feelings of psychological safety around her co-teachers:

“I feel like if they find out I didn’t follow them [in changing the grades], it will mean war. It’s like … you know, I’ll be the one they’ll always see and pick on. That’s really my fear whenever I make reports.”

On the other hand, *gaan* was experienced when teachers felt that their asserted *pagdadala* was righteous, justified and validated. For instance, Teacher Elaine remarked how providing care in ways she thought were righteous helped foster calm for her, despite backlash from others:

“Though I can still hear them say ‘*bida-bida* (eager beaver)’ there, ‘Do what you will, report it … wash your hands off us.’ But now my heart is calm, unlike last year. Maybe because I can see that I’m walking the right path.”

Seeing their asserted caring ways positively impact students eventually made teachers feel that their troubles were worth it. As Shay shared:

“Even if you go through situations that were, let’s say … unacceptable to child protection [policy], but just look at how they eventually turned out ok. Their lives are ok.”

Finding supportive groups of peers or mentors (e.g., co-teachers, supervisors, colleagues from religious organizations) who shared their principles also helped teachers find meaning and purpose in taking a stand. Holding onto hopes about future positive changes in systems they perceived as unhelpful also lead to *gaan*. These opportunities for *gaan* can be accessed in spaces of *pahinga* where teachers felt their sentiments and hopes for change in systems are heard, understood, acted upon or sustained. These spaces included nooks in the classroom where beholding students reminded teachers of their sense of purpose, chat boxes with like-minded peers or offices of supportive mentors or more knowledgeable others.

“Every time that I start to get tired, every time I feel that I can’t do it anymore, that’s when we’ll just chat each other up, strengthening each other … actually we have a separate group chat. Because it’s there where I really feel that what I do makes sense.” Teacher Elaine

### *Caring along inaccessible paths: curtailed* Pagdadala

Narratives of curtailed *pagdadala* featured teachers whose attempts of bearing their caring responsibility were significantly limited or cut short by the loss of their formal adviser-student relationship prior to any significant resolution of the caring narrative. Students moved onto inaccessible spaces of care outside the classroom where the teacher’s influence and formal accountability over them is gone. Teachers identified themselves as limited or unhelpful co-burden-bearers because their aspired *patutunguhan* for the student were left unrealized. Experiences of *bigat* in curtailed *pagdadala* related with feelings of grief over their student’s circumstances and grappling with one’s limitation as a co-burden bearer. *Gaan* was related with acceptance of one’s limitations and hope for better outcomes for students.

#### “I couldn’t do anything”

Curtailed *pagdadala* is illustrated in Teacher Jenny’s story of caring for Jong, her sixteen-year-old grade six student who was accused of rape and taken to a juvenile centre in the middle of the school year. Teacher Jenny described herself as a strict but compassionate teacher who endeavoured to shape her student’s character. She initially thought that Jong’s long absence was merely due to his being a breadwinner, as he worked in a factory to provide for his family. However, she found out a month later that he was already being detained. She visited him in the Juvenile Center, attempting to comfort, listen and uplift him, even while she herself grappled with her own grief over Jong’s misfortune. She held back her tears the whole time, attempting to be a source of strength for Jong.

Up until the time of the interview, Teacher Jenny was still awaiting what would happen to Jong and was anxious about his future. She wanted to confront the parent who filed the rape case but was advised by the school to not get involved, as it was already under the jurisdiction of the Department of Social Welfare and Development. Reflecting on Jong’s case, Teacher Jenny shared about her shock regarding her students’ increasingly complex burdens and her thoughts on its links to larger community concerns:

“It’s disturbing, because … But if you think about it, it’s also in the community. As a whole. There are many factors why we are like this or why the students are in these situations. Because of technological progress, in the laws made by … lawmakers—and maybe the most [contributing factor] is the systems of education, if only we can fix it.”

#### Inaccessible spaces of care

*Pagdadala* in caring was curtailed when students stopped showing up for class due to drop-outs, transfers or promotions to the next level. Reasons for their disappearance included family movements, disengagement, getting hooked on vices, illegal substance or out-of-school peers, being family breadwinners, financial incapacity or having legal cases. Teachers were able to provide support in varying degrees—some narratives began right when the students were already missing while others were about attempts to provide shared, overextended or asserted *pagdadala* that was cut short. Afterwards, teachers encountered or heard about their lost students from inaccessible spaces of care—in classrooms of other teachers or other schools, on the streets sniffing rugby, in relocation sites of other cities, etc. In these inaccessible spaces, the teachers’ proximity and influence over the student is diminished or completely gone, limiting the teachers’ co-burden bearing efforts. Jenny and Queena shared their attempts to reach out to their students in these inaccessible spaces of care:

“When we visited him [Jong], he was the only one opening up to us because we were prohibited to interview because the DSWD [case worker] was right there in front of us. We couldn’t really talk in private.” Teacher Jenny

“I have another student who stopped coming to school because he had many siblings, thirteen and he was the youngest, and his mom was in prison because of *tokhang*. I went to their house and that’s when I found out that he no longer had parents.” Teacher Queena

This was also the case for Teacher Vanessa who tried to help Nel, her former first grader who was touching her genitalia repeatedly in class. Vanessa tried talking to Nel, consulting with the guidance teacher, and calling for Nel’s unresponsive parents. However, Nel continued her behaviour until the school year ended. Feeling like she failed Nel, Vanessa shared how she tried looking for her next school year:

“I tried to find which class adviser will handle her next. I asked about her because I had a friend teaching Grade 2. [I was told] She wasn’t schooling here anymore.”

#### Bigat *and* gaan *in loss*

*Bigat* in curtailed *pagdadala* highlighted feelings of loss, grief, regret and guilt over losing students or opportunities to help out. Students’ misfortunes inspired feelings of pity, questioning of why things happened, and anger or hopelessness towards perceived causes of students’ suffering. Losing students also led to feelings of failure, lowered sense of competence or guilt over being unhelpful—which teachers carried with them even years after the events happened. In the words of Teacher Vanessa:

“[Every time I talk about Nel] you know there’s this, this, guilt. There. It’s like, I wasn’t even able to help or wasn’t able to save her, if I had to save her.”

On the other hand, *gaan* was related with gradually accepting one’s limitations and boundaries as teacher-carer, and finding ways to sustain hope for better outcomes for students. Limit acceptance included acknowledging the societal factors influencing students’ lives beyond their control and identifying areas they can influence. Teachers shifted their mindset from “why did this happen?” to “what can I still do?” or channelled their efforts back to their remaining students. Hopes for students’ better welfare were reinforced in rare occasions when teachers hear about former students faring better. Teacher Queena shared about how she came to terms with losing a student by focusing on her area of control:

“Maybe at first, it was hard and I carried that with me. (…) But then I remembered a time when I couldn’t provide for all, so whatever I could give in the classroom, whatever I can muster to give there, I give. That’s how I make up for it. I guess the world, the world failed them. So I don’t want that to happen in the school too.”

For this pathway, spaces of *pahinga* included ones that helped teachers ventilate or re-story their narratives in ways that allowed for *gaan*. These could be in private reflective spaces on their own, inside the classrooms as they beheld their remaining students, or in the presence of a listening other. As Jenny shared:

“It’s not really necessary that if you have a problem, somebody gives the solution. That’s

not what we teachers need. We need to release our problems. (…) To be able to really share our stories with someone.” Teacher Jenny

## Discussion

Most literature on caring and its costs for educators focus on the psychosocial distresses of supporting burdened students within school spaces. They also hail from school contexts where there is higher access to specialized psychosocial support manpower, programmes and material resources. By using Clandinin’s narrative inquiry framework complimented by the cultural model of *Pagdadala*, this study contributes a narrative map of teacher care work that accounts for the socio-cultural, spatial and temporal contexts of low-resource schools. Resulting *pagdadala* pathways reflect a storied landscape of caring where teacher’s *pagdadala* narratives unfolded alongside narratives of students, other carers and institutions of care across home, school and community spaces through time. These pathways portray teachers’ roles, goals and ways in co-burden-bearing as dynamically shaped by the confluence of multiple narratives within the caring landscape. And that, as teachers engage in the emotional labour of care, experiences of *bigat* may occur alongside experiences of *gaan* through time and relates to more than just witnessing student distress.

This study also contributes empirical grounding for the utility of Decenteceo’s *Pagdadala* model in understanding teachers’ caring work and its costs in urban poor contexts, as well as guide ways of supporting teacher-carers. Theoretical frameworks on culturally-sensitive counselling approaches emphasize the need to consider the client’s language as well as the context of the larger relational and oppressive structures in conceptualizing and addressing their concerns (Atkinson et al., 1993; Comstock et al., 2008; Ridley et al., 1994). Findings provide evidence for how *Pagdadala*’s locally-familiar metaphorical language can lend ease for teacher clients to share their stories, consistent with endorsements from prior *pagdadala*-related studies (Landoy et al., 2015; Noguera, 2013). The pathways also illustrate how social and structural contexts of low-resource schools influence culture-specific challenges and rewards for lay helpers. Meanwhile, what other theoretical frameworks and studies do not feature, but results highlight, are the empirical narrative accounts of how a cultural-story model can frame carer experiences with consideration to their everyday contexts. Although we recognize that professional psychologists and teachers have distinct yet overlapping roles in forming the lives of students in educational institutions, findings further emphasize the constitutive role of everyday material and spatial spaces in shaping the ethical dimension of caring for others as well as themselves.

### *Caring for burdened students through the lens of* Pagdadala

Findings illustrate how teachers’ *pagdadala* of care echo ways of caring cited in literature such as the extension of personal, academic, socio-emotional and physical care aimed at supporting burdened students’ wellbeing and academic development. On the other hand, findings also highlight the salient and unique ways by which teachers emphasized the more material, social and collaborative aspects of bearing their caring responsibilities. Beyond supporting current wellbeing and academic needs, the teachers’ *patutunguhan* of their *pagdadala* of care across pathways include the ability to connect with their students and contribute to better their state of life in the future. Towards these ends, teachers take on a variety of roles as *taga-dala* and employ multiple or varied ways of caring they deem helpful to achieve these *patutunguhan*. In contexts where student poverty and lack of resources ispart of everyday reality, teachers employing overextended *pagdadala* emphasize providing care via material support and adopting supplementary caring roles). In providing socio-emotional care, teachers leverage on the cultural Filipino interpersonal value of *pakikiramdam* or shared-inner-perception as a tool to assess distress and navigate caring interactions with students. Lastly, findings highlight teachers’ leveraging greatly on collaborating with other carers, especially parents and guardians, in caring for distressed students.

The empirical narrative accounts of *pagdadala* also lend insight in how educators actively define the elements of their *pagdadala* stories in caring. Decenteceo posited that it is the community which primarily defines the elements of *Pagdadala* and the individuality of the *taga-dala* is deemphasized. Findings suggest, however, that teachers as co-burden-bearers actively negotiate personally aspired *pagdadala* pathways vis-à-vis the multiple and sometimes conflicting pathways defined in low-resource school communities. Daisy, Bhe, Shay and Jenny spoke of aspired *pagdadala* pathways where they anchored their roles, ways of caring and *patutunguhan* on their personal histories, ethos and expectations about care work. Because of her SPED background, Daisy’s aspired pathway involved identifying as a SPED advocate who enabled students’ potential to give them a chance at life. Meantime, as one who credits her life success to her strict upbringing, Shay’s aspired pathway involved being a caring but strict teacher who is willing to risk her licence for the sake of setting students on the right path.

On the other hand, the extent to which teachers were able to live out their aspired *pagdadala* is influenced by other dominant narratives within the caring landscape. Home spaces hold the lived *pagdadala* of parental figures as co-burden-bearers, which in turn, shape the *pagdadala* stories that students live and tell in schools. School spaces prescribe pathways of *pagdadala* that is institutionalized in care-related policies or embodied in lived practices of co-teachers. This includes legally mandated roles of Filipino teachers as *in loco parensis* who employ CPP or mental health policy aligned caring practices towards holistically developing young citizens. As illustrated in overextended *pagdadala*, institutional narrative of care also included the practice of assigning multiple caring roles to augment lack of manpower. The pathways illustrate the varying degrees to which these other caring narratives can shape the teacher’s lived narratives by enabling or hindering their aspired ones. They reflect the overlapping influence of homes, schools and communities over child care (Epstein, 2009), and underscore how distresses in care work in urban poor settings maybe inseparable from its socio-cultural, material and structural contexts.

### *Costs of caring in the language of* bigat-gaan

The experiences of *bigat* in teachers’ *pagdadala* support findings from previous literature on how exposure to the distresses of students may pose a range of cognitive and emotional costs for carers. On the other hand, examining the cost of caring through the language of *bigat-gaan* shows how experiences of ease or *gaan* is also possible alongside experiences of *bigat*. Findings illustrate *bigat-gaan* as present across all narrative pathways and relate it with the teachers’ subjective evaluation of the *dinadala*’s importance, as well as the sense of ease with which they are able to live out their aspired *pagdadala*. These support Decenteceo’s conceptualization of *bigat* as experienced when encountering challenges in *pagdadala*. As a *dinadala*, care work is subjectively evaluated by teachers as *mabigat* in terms of its importance, invoking the imagery of caring as “holding the students’ life” (e.g., *“Dala mo ang buhay”*). Hence, to bear caring as a responsibility is not taken lightly, leading to teachers’ exerting internal pressure upon themselves to do it well and consequently taking it hard when they fail. This weighty valuing and sense of accountability attached to care reflects the wider cultural narrative of Filipino educators of seeing teaching as a way to transform lives, especially in low-resource contexts where education is seen as students’ ticket out of poverty (Arzadon, 2016; Quejada & Orale, 2018; Rogayan, 2018). This is further reinforced by systems that hold teachers legally accountable for students under their care.

Findings also suggest *bigat-gaan* experiences to relate with the perceived level of ease of the process of living out one’s aspired narrative, as well as the perceived quality of the outcome of one’s *pagdadala*. For instance, shared *pagdadala* was the pathway where teachers’ aspired narratives were both most enabled and successfully completed. Sharing congruent pathways with co-burden-bearers or caring systems not only enable teachers to live out their preferred roles, ways and goals of caring, but also delegate caring responsibilities in a way that facilitates a great sense of ease in completing *pagdadala*. Hence, even while still considered *mabigat* as a responsibility, caring also became *magaan—*easier and rewarding—because of the sense of connection, support and accomplishment that teachers experienced. On the other hand, conflicting pathways that hindered or undermined the teachers’ aspired one within overextended, asserted and curtailed *pagdadala* led to varied psychosocial, material and physical experiences of *bigat*. Witnessing students’ sufferings and being unable to help, a narrative in stark contrast with teachers’ aspired one, gave rise to emotional experiences of *bigat* as shock, great pity, helplessness and sense of incompetence. Conflicting caring narratives of other carers and unsupportive narratives of policies and practices, led to the *bigat* of social conflict, threats to personal dignity, heavier workloads, material and physical strain, and others.

These findings suggest that the less congruent the teachers’ lived narratives were vis-à-vis their aspired ones, the more likely they experience greater *bigat* in care work. This partially echoes how, in teachers’ emotional labour in caring, distress is related to teachers’ sense of dissonance when the outward expression of one’s caring emotions is in conflict with what one genuinely feels (Wang et al., 2019). It may also imply that *bigat* in *pagdadala* in low-resource contexts is not only influenced by exposure to the student’s suffering per se (as in compassion fatigue), but also by the social, material, and systemic barriers that teachers encounter in negotiating the kind of care they aspire to provide. Notably, emotional labour literature also points to re-appraisal, or adoption of different perspectives of situations, as helpful in changing feeling about situations (Mesquita & Delvaux, 2013). This was partially supported by teachers with curtailed narratives who initially grappled with the *bigat* of loss but were able to re-appraise their locus of control that led to the sense of *gaan* in accepting their loss.

### *Cultivating teacher wellbeing via spaces of shared* pagdadala *and* pagpapahinga

Findings point to the potential help of cultivating spaces of shared *pagdadala* and *pagpapahinga* in enabling greater *gaan* and higher success in care work. Given the amplified challenges and psychosocial costs of care in low-resource contexts, cultivating these spaces may promote greater wellbeing for teachers and, by extension, for students too. Shared *pagdadala* can be cultivated in affiliative spaces of care where teachers align their pathways (roles, goals and ways of caring) with other co-burden-bearers. These maybe physical spaces (e.g., home, schools) or online spaces (parent-teacher chat boxes) where carers come together to connect, identify, and delegate caring efforts for student welfare. Findings also suggest the helpfulness of dedicated spaces where teachers themselves can receive specialized support in their *pagdadala*, most especially when guardians are inaccessible or student cases are beyond their expertise, as portrayed in overextended and curtailed *pagdadala*. This may be in mentoring or peer-supported physical or online spaces with senior teachers, supervisors, guidance counsellors, and other child-care specialists.

Supporting Decenteceo’s conceptualization of *pahinga*, spaces of *pagpapahinga* illustrated in the pathways are those which enable teachers to distance themselves from their responsibilities, physically rest and recuperate, and share stories with peers. These spaces enable teachers’ meaning-making on the purpose of their work, celebrate success, and reflect on the congruence of their aspired and lived *pagdadala* narratives. They also foster connectedness with co-burden-bearers, enabling teachers to savour being with students inside classrooms and bond with parents or co-teachers. Access to these spaces in low-resource schools can be limited by the lack of awareness of caring’s costs and insufficient material and human resources to cultivate such spaces. Therefore, interventions aimed to support teacher-carers may consider increasing awareness on care work’s risks and increasing access to these spaces. This may include implementation of care-related policies in a way that gives due consideration to its risk for teachers and strengthening provisions for supportive mechanisms that promote *gaan*, shared *pagdadala*, and access to spaces of *pagpapahinga*.

Understanding care work and its costs for educators through *Pagdadala* also provides information for helping professionals who wish to integrate this story-model in wellbeing interventions for teacher carers. Findings of this study provide support how the language of the *pagdadala* model can be used by teachers in narrating their caring experiences and concerns. Aside from promoting shared *pagdadala* and *pagpapahinga*, findings point to the potential of helping teachers compose *pagdadala* stories that help them achieve a sense of congruence between their aspired and lived caring narratives, a sense of connection with their students and other carers, and a sense of accomplishment in reaching their caring goals. Furthermore, findings underscore the importance of attending not just to the intrapersonal context of teachers or distress of students, but also to the wider social, material, and structural challenges of their care work. This echoes how individual counselling issues in the Philippine setting are typically reflections of wider societal problems that set the parameters for how change and wellbeing can be fostered (Tuason et al., 2012).

### Limitations

The usefulness of metaphors in understanding and articulating narrative experiences is limited by cultural and linguistic familiarity of those using them (Decenteceo, 1999). Hence, results of this study may be limited due its small sample size. Furthermore, the conceptualization of care and co-burden-bearing may also differ across different carer and gender roles, tenure, and socio-economic context (Coultas et al., 2016).

### Implications

Results of this study may be most relevant for teachers with similar demographic profile and degree of familiarity with metaphors of burden-bearing. Future studies with greater sample sizes of teachers who are non-female, non-advisers, tenured and those who teach in rural areas, may strengthen evidence for the utility of the *pagdadala* model for carers in diverse education settings.

### Conclusion

In summary, the key findings of the study highlighted four narrative pathways of *pagdadala* of caring that teachers lived and told across the caring landscape: shared, overextended, asserted, and curtailed. Complimenting literature on care work in education using Clandinin’s narrative inquiry framework, this study has offered a storied map of co-burden-bearing that was shaped by the social, spatial and temporal contexts in low-resource urban public schools by integrating Decenteceo's*Pagdadala* model. The results and discussion show how teachers lived their pathways of *pagdadala* by actively negotiating their aspired caring narratives vis-à-vis the concurrent *pagdadala* narratives as lived and prescribed by students, other carers, and caring systems. Theoretical and practical implications highlight the dynamics of *bigat-gaan* in care work and the potential advantage of leveraging on shared *pagdadala* and spaces of *pagpapahinga* in supporting teacher wellbeing.

## Notes

1. Teachers (without guidance licence) who provides guidance services and serves as the main guidance coordinator for schools.

2. A single teacher holds the whole classroom for the whole duration. This is done for Kinder, Grades 1 to 3. By the 4th grade, teachers start to shift from class to class per subject.

## References

[cit0001] Abraham-cook, S. (2012). The prevalence and correlates of compassion fatigue, compassion satisfaction, and burn-out among teachers working in high-poverty urban public schools [Doctoral dissertation]. Proquest LLC. https://search.proquest.com/docview/1461391768

[cit0002] Acton, R., & Glasgow, P. (2015). Teacher wellbeing in neoliberal contexts : A review of the Literature. *Australian Journal of Teacher Education*, 40(8), 99–114. 10.14221/ajte.2015v40n8.6

[cit0003] Alisic, E. (2012). Teachers’ perspectives on providing support to children after trauma: A qualitative study. *School Psychology Quarterly*, 27(1), 51–16. 10.1037/a0028590 22582936

[cit0004] Alisic, E., Bus, M., Dulack, W., Pennings, L., & Splinter, J. (2012). Teachers’ experiences supporting children after traumatic exposure. *Journal of Traumatic Stress*, 25(1), 98–101. 10.1002/jts.20709 22354512

[cit0005] Arzadon, M. M. C. E. (2016). Where machines rant, recite poems, and take outrageous selfies: An ethnography of a teachers’ facebook group. *Studies in Educational Ethnography*, 13, 65–96. 10.1108/S1529-210X20150000013003

[cit0006] Atkinson, D., Thompson, C., & Grant, S. (1993). A three-dimensional model for counseling racial/ethnic minorities. *The Counseling Psychologist*, 21(2), 257–277. 10.1177/0011000093212010

[cit0007] Brunzell, T., Stokes, H., & Waters, L. (2018). Why do you work with struggling students? Teacher perceptions of meaningful work in trauma-impacted classrooms. *Australian Journal of Teacher Education*, 43(2), 116–142. 10.14221/ajte.2018v43n2.7

[cit0008] Clandinin, D. J., Caine, V., Lessard, S., & Huber, J. (2016). *Engaging in narrative inquiries with children and youth*. Routledge.

[cit0009] Comstock, D. L., Hammer, T. R., Strentzsch, J., Cannon, K., Parsons, J., & Gustavo Salazar, I. I. (2008). Relational-cultural theory: A framework for bridging relational, multicultural, and social justice competencies. *Journal of Counseling and Development*, 86(3), 279–287. 10.1002/j.1556-6678.2008.tb00510.x

[cit0010] Coultas, C., Broaddus, E., Campbell, L. A., Mutsikiwa, A., Madanhire, C., Nyamukapa, C., & Gregson, S. (2016). Implications of teacher life–work histories for conceptualisations of ‘care’: Narratives from rural Zimbabwe. *Journal of Community & Applied Social Psychology*, *26*(2), 323–339. https://doi.org/10.1002%2Fcasp.2265 27499602 10.1002/casp.2265 PMC4950062

[cit0011] Decenteceo, E. T. (1996). “Burden-bearing” as a metaphor for counselling: Experiences from the Philippines. *Intercultural Pastoral Care and Counselling*, 1, 30–31. http://www.federschmidt.net/workbook/workbook-final1.pdf

[cit0012] Decenteceo, E. T. (1997). *Rehab: Psychosocial rehabilitation for social transformation; Some programs and concepts*. Aeon Printers.

[cit0013] Decenteceo, E. T. (1999). The *Pagdadala* model in counseling and therapy. *Philippine Journal of Psychology*, 32(2), 89–104. https://pssc.org.ph/wp-content/pssc-archives/

[cit0014] Denise Valdez, V. (2018, 1 14). Lack of registered guidance counselors forces schools to keep unlicensed ones (Part 1). *ABS-CBN News*. https://news.abs-cbn.com/focus/01/14/18/lack-of-registered-guidance-counselors-forces-schools-to-keep-unlicensed-ones-part-1

[cit0015] Department of Education. (2015, 5 29). Teaching loads and assignments of public school teachers (DepEd-NCR Memorandum No. 105 s. 2015). *TeacherPh*. https://www.teacherph.com/teaching-loads/

[cit0016] Figley, C. R. (1995). Compassion fatigue as secondary traumatic stress disorder: An overview. *Compassion Fatigue: Coping with Secondary Traumatic Stress Disorder in Those Who Treat the Traumatised*, (23), 1–20. http://books.google.com/books?hl=en&lr=&id=2Cwo47uOEq4C&pgis=1

[cit0017] Franklin, C., Kim, J. S., Beretvas, T. S., Zhang, A., Guz, S., Park, S., … Maynard, B. R. (2017). The effectiveness of psychosocial interventions delivered by teachers in schools: A systematic review and meta-analysis. *Clinical Child and Family Psychology Review*, 20(3), 333–350. 10.1007/s10567-017-0235-4 28493176

[cit0018] García-Carmona, M., Marín, M. D., & Aguayo, R. (2019). Burnout syndrome in secondary school teachers: A systematic review and meta-analysis. *Social Psychology of Education: An International Journal*, 22(1), 189. 10.1007/s11218-018-9471-9

[cit0019] Geronimo, J. Y. (2013, 11 28). How schools can help kids heal from disaster trauma. *Rappler*. https://www.rappler.com/move-ph/issues/disasters/typhoon-yolanda/44833-schools-therapy-disaster-trauma-haiyan

[cit0020] Gu, Q., & Day, C. (2013). Challenges to teacher resilience: Conditions count. *British Educational Research Journal*, 39(1), 22–44. 10.1080/01411926.2011.623152

[cit0021] Hill, A. C. (2011). *The cost of caring: An investigation in the effects of teaching traumatized children in urban elementary settings*. [Doctoral dissertation] https://scholarworks.umass.edu/open_access_dissertations

[cit0022] Jennings, P. A., & Greenberg, M. T. (2009). The prosocial classroom: Teacher social and emotional competence in relation to student and classroom outcomes. *Review of Educational Research*, 79(1), 491–525. 10.3102/0034654308325693

[cit0023] Kim, J. (2016). *Understanding narrative inquiry*. Sage Publications, Inc.

[cit0024] Koenig, A., Rodger, S., & Specht, J. (2018). Educator burnout and compassion fatigue: A pilot study. *Canadian Journal of School Psychology*, 33(4), 259–278. *Koenig*. 10.1177/0829573516685017

[cit0025] Landoy, B. V., Hechanova, M. R., Ramos, P. A., & Kintanar, N. S. (2015). The application and adaptation of psychological first aid: The Filipino psychologists’ experience after Typhoon Haiyan. *Philippine Journal of Psychology*, 48(2), 81–104. https://archium.ateneo.edu/psychology-faculty-pubs/31/

[cit0026] Lei, H., Cui, Y., & Chiu, M. M. (2018). The relationship between teacher support and students’ academic emotions: A meta-analysis. *Frontiers in Psychology*, 8, 1–12. 10.3389/fpsyg.2017.02288 PMC5786576 29403405

[cit0027] Lovibond, S. H., & Lovibond, P. F. (1995). *Manual for the depression anxiety stress scales* (2nd ed.). Psychology Foundation.

[cit0028] Malipot-Hernando, M. (2018, 8). DepEd urged to reduce teachers’ workload. *Manila Bulletin*, https://news.mb.com.ph/2018/08/02/deped-urged-to-reduce-teachers-workload/

[cit0029] Mesquita, B., & Delvaux, E. (2013). A cultural perspective on emotion labor. In A. Grandey, J. Diefendorff, and D. Rupp (Eds.). Emotional Labor in the 21st Century: Diverse Perspectives on Emotion Regulation at Work, pp. 251–272. Routledge.

[cit0030] Montgomery, C., & Rupp, A. (2005). A meta-analysis for exploring the diverse causes and effects of stress in teachers. *Canadian Journal of Education/Revue Canadienne De L’éducation*, 28(3), 458. 10.2307/4126479

[cit0031] Noddings, N. (1992). *The challenge to care in schools: An alternative approach to education*. Teachers’ College Press.

[cit0032] Noguera, R. T. (2013). The narratives of children in armed conflict: An inference to spirituality and implication to psychological intervention. *International Journal of Children’s Spirituality*, 18(2), 162–172. 10.1080/1364436X.2012.755954

[cit0033] Official Gazette of the Republic of the Philippines. (2019). *Implementing rules and regulations of republic act no. 11036*. https://www.officialgazette.gov.ph/2019/01/22/implementing-rules-and-regulations-of-republic-act-no-11036/

[cit0034] Olores-Regalado, M. (2017). Career mobility and gender: A descriptive study of selected DepEd teachers in Iligan city. In S. Aimimtham & A. Nurmandi (Eds.), *Complexity in managing local governments in selected Asean countries* (pp. 139–162). Asia Pacific Society for Public Affairs.

[cit0035] Pagayanan, R. P. (2016). *Stress Profile of Public Elementary School Teachers in Tacloban City Division: Inputs for a Proposed Classroom Intervention Program*. 2013–2016. 10.17758/uruae.uh0516121

[cit0036] Quejada, A., & Orale, R. (2018). Lived experiences of elementary teachers in a remote school in Samar, Philippines. *Journal of Academic Research*, 3(3), 1–13. https://jar.ssu.edu.ph/index.php/JAR/article/view/7

[cit0037] Ridley, C. R., Mendoza, D. W., Kanitz, B. E., Angermeier, L., & Zenk, R. (1994). Cultural sensitivity in multicultural counseling: A perceptual schema model. *Journal of Counseling Psychology*, 41(2), 125–136. 10.1037/0022-0167.41.2.125

[cit0038] Rogayan, J. D. (2018). Why young Filipino teachers teach? *Asia Pacific Higher Education Research Journal*, 5(2), 48–60. http://po.pnuresearchportal.org/ejournal/

[cit0039] Schaefer, L., & Clandinin, J. D. (2018). Sustaining teachers’ stories to live by: Implications for teacher education. *Teachers and Teaching*, 25(1), 54–68. 10.1080/13540602.2018.1532407

[cit0040] Tomacruz, S. (2019). A cry for help: Mental illness, suicide cases rising among youth. *Rappler*. https://www.rappler.com/newsbreak/in-depth/211671-suicide-cases-mental-health-illness-youth-rising-philippines

[cit0041] Topp, C. W., Østergaard, S. D., Søndergaard, S., & Bech, P. (2015). The WHO-5 well-being index: A systematic review of the literature. *Psychotherapy and Psychosomatics*, *84*(3), 167–176. 10.1159/000376585 25831962

[cit0042] Tuason, M. T. G., Galang Fernandez, K. T., Catipon, M. A. D. P., Trivino-Dey, L., & Arellano-Carandang, M. L. (2012). Counseling in the Philippines: Past, present, and future. *Journal of Counseling and Development*, 90(3), 373–377. 10.1002/j.1556-6676.2012.00047.x

[cit0043] United Nations – Habitat (2003). The challenges of slums: Global report on human settlements 2003. *United Nations*. https://www.un.org/ruleoflaw/blog/document/the-challenge-of-slums-global-report-on-human-settlements-2003/

[cit0044] Wang, H., Hall, N. C., & Taxer, J. L. (2019). Antecedents and consequences of teachers’ emotional labor: A systematic review and meta-analytic investigation. *Educational Psychology Review*, 31(3), 663–698. 10.1007/s10648-019-09475-3

[cit0045] Wentzel, K. R. (1997). Student motivation in middle school: Role of perceived pedagogical care. *Journal of Educational Psychology*, 89(3), 411–419. 10.1037/0022-0663.89.3.411

[cit0046] Wentzel, K. R. (1998). Social relationships and motivation in middle school: The role of parents, teachers and peers. *Journal of Educational Psychology*, 90(2), 202–209. 10.1037/0022-0663.90.2.202

[cit0047] Wessels, E., & Wood, L. (2019). Fostering teachers’ experiences of well-being: A participatory action learning and action research approach. *South African Journal of Education*, 39(1), 1–10. 10.15700/saje.v39n1a1619

[cit0048] Ziaian-Ghafari, N., & Berg, D. (2019). Compassion fatigue: The experiences of teachers working with students with exceptionalities. *Exceptionality Education International*, 29(1), 32–53. 10.5206/eei.v29i1.7778

